# Cytomegalovirus infection in ulcerative colitis assessed by quantitative polymerase chain reaction: risk factors and effects of immunosuppressants

**DOI:** 10.3164/jcbn.18-14

**Published:** 2018-06-08

**Authors:** Yujiro Henmi, Kazuki Kakimoto, Takuya Inoue, Kei Nakazawa, Minori Kubota, Azusa Hara, Takashi Mikami, Yutaka Naka, Yuki Hirata, Yoshimasa Hirata, Taisuke Sakanaka, Sadaharu Nouda, Toshihiko Okada, Ken Kawakami, Toshihisa Takeuchi, Kazunari Tominaga, Kazuhide Higuchi

**Affiliations:** 1 2nd Department of Internal Medicine, Osaka Medical College, 2-7 Daigakumachi, Takatsuki, Osaka 569-8686, Japan

**Keywords:** ulcerative colitis, cytomegalovirus, polymerase chain reaction, endoscopic features, tacrolimus

## Abstract

We investigated the risk factors of and appropriate treatment for cytomegalovirus colitis in patients with ulcerative colitis, using quantitative polymerase chain reaction analysis to detect cytomegalovirus in the colonic mucosa. Between February 2013 and January 2017, patients with exacerbated ulcerative colitis who were admitted to our hospital were consecutively enrolled in this retrospective, single-center study. Patients were evaluated for cytomegalovirus using serology (antigenemia) and quantitative polymerase chain reaction analyses of the colonic mucosa, which were sampled during colonoscopy. Of 86 patients, 26 (30.2%) had positive quantitative polymerase chain reaction results for cytomegalovirus; only 4 were also positive for antigenemia. The ages of the cytomegalovirus DNA-positive patients were significantly higher than those of negative patients (*p* = 0.002). The mean endoscopic score of cytomegalovirus DNA-positive patients was significantly higher than that of cytomegalovirus DNA-negative patients. Treatment with combined immunosuppressants was associated with an increased risk of cytomegalovirus. Fourteen of 15 (93.3%) cytomegalovirus DNA-positive patients who were negative for antigenemia showed a clinical response to treatment with additional oral tacrolimus, without ganciclovir. cytomegalovirus reactivation in active ulcerative colitis is associated with age and combined immunosuppressant therapy. Because additional treatment with tacrolimus was effective, patients who are negative for antigenemia and cytomegalovirus DNA-positive colonic mucosa may recover without antiviral therapy.

## Introduction

Cytomegalovirus (CMV) infection has been associated with refractory ulcerative colitis (UC).^(1,2)^ Moreover, it can complicate severe steroid-resistant UC.^(3,4)^ Several different methods such as histology with immunohistochemistry and serology, including an antigenemia assay, have been used to diagnose CMV infection in patients with UC. However, these diagnostic techniques were not reported to have sufficient sensitivity for detecting CMV infection.^(5)^ Therefore, diagnosing complicated CMV infection in patients with UC remains difficult.^(6)^

Quantitative polymerase chain reaction (qPCR) has shown to have high sensitivity for detecting CMV infection in the colonic mucosa.^(5)^ Yoshino *et al.*^(7)^ demonstrated the utility of the mucosal PCR method for diagnosing CMV infection in patients with active UC. However, the high sensitivity of qPCR may result in low specificity for diagnosing active CMV infection because CMV DNA with a low copy number would be detected by qPCR but may not actually reflect active infection.^(8)^ CMV is frequently reactivated in patients with UC, even in those without clinical symptoms and high CMV antigen concentrations.^(9)^ Several studies have reported that CMV infection is not associated with worsening of the inflammatory activity of the intestines.^(9,10)^ Therefore, the appropriate therapeutic approach for UC patients with CMV DNA-positive colonic mucosa remains to be determined.

Several reports suggested that commonly used immunosuppressive therapies, including steroids, were associated with a high risk of CMV infection in patients with UC.^(2,6)^ However, the etiology of active CMV colitis in patients with UC has not been clarified. Of note, the potent immunosuppressant agent tacrolimus, which has been shown to be safe and effective as a salvage therapy for refractory cases of UC.^(11–13)^ has been reported to decrease the incidence of viral infections in liver transplant recipients.^(14–16)^ To our knowledge, this is the first report to evaluate the effects of tacrolimus on the number of CMV DNA copies in the colonic mucosa of patients with UC exacerbation and concomitant CMV infection.

We conducted this study to identify medications that are associated with CMV colitis during the treatment of UC, the role of endoscopy for diagnosing CMV colitis, and the appropriate therapeutic approach for CMV colitis in patients with exacerbated UC, using a qPCR analysis to detect CMV infection in the colonic mucosa.

## Materials and Methods

### Study design and patients

This restrospective, observational, single-center study was approved by the Ethical Committee of Osaka Medical College. Between February 2013 and January 2017, patients admitted to the Osaka Medical College Hospital, Osaka, Japan, with UC exacerbation were consecutively enrolled. The inclusion criteria were as follows: (1) a UC diagnosis that was established according to standardized criteria, based on prior clinical, radiologic, endoscopic, and histologic findings; (2) total colonoscopy with colonic mucosa samples that were obtained for the qPCR analysis, to diagnose CMV infection; and (3) serology (antigenemia) that was performed on admission. The extent of colonic involvement was determined using total colonoscopy. Patients were divided into four groups based on the prescribed medications on admission, as follows: 5-aminosalicylic acid (5-ASA) alone; prednisolone (PSL); immunosuppressant monotherapy with tacrolimus, biologics, or thiopurines (monotherapy); and combination immunosuppressant therapy that included PSL.

### Diagnosis of CMV infection

All patients who were enrolled to this study were evaluated for the presence of CMV, using antigenemia testing of blood samples. These patients underwent qPCR testing of inflamed colonic mucosa obtained during the colonoscopy that was performed on admission. The CMV antigenemia assay results are expressed as the number of CMV antigen-positive cells per 50,000 leukocytes, and a positive result for the CMV antigenemia assay was defined as one or more CMV-positive cells per 50,000 leukocytes. According to a report by Yoshino *et al.*^(7)^ qPCR for CMV DNA detection in the colonic mucosa was performed. The biopsy was performed from the most inflamed points in the colon. Patients whose specimens contained >10 CMV DNA copy numbers per µg of DNA were considered positive for CMV infection.^(7)^ The positive results of any test were considered to indicate CMV infection.

### Endoscopic scores and features

**Fig. 1:**
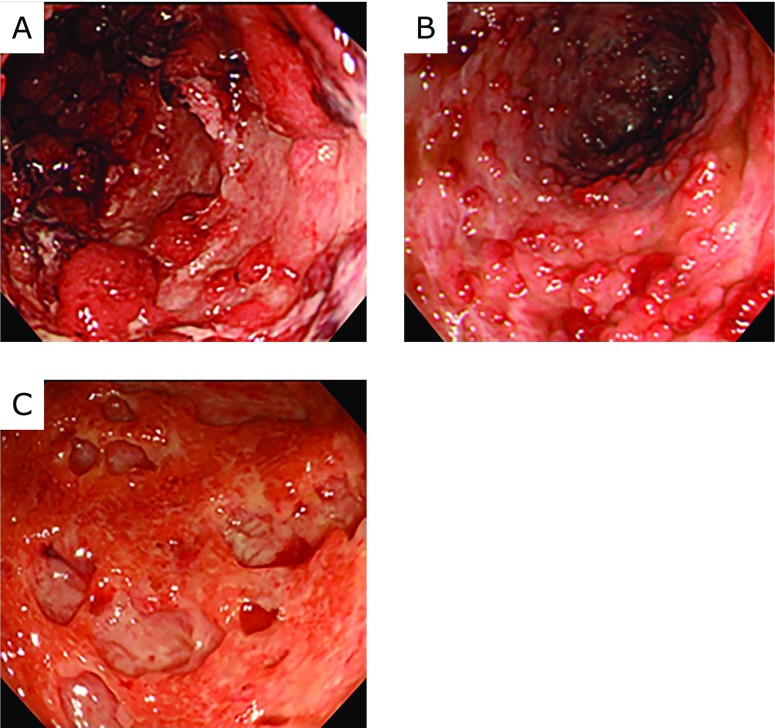
Typical endoscopic findings of ulcerative changes. (A) Longitudinal ulcers, (B) Wide mucosal defects, (C) Punched-out ulcers.

The disease severity on admission was scored based on the endoscopic index of Blackstone, which was defined as the endoscopic score in this study.^(17,18)^ Additionally, endoscopic features were evaluated by examining the colonoscopic images. According to previous reports,^(19,20)^ as shown in Fig. 1, we determined whether the following forms of ulceration were present: longitudinal ulcers, wide mucosal defects, and punched-out ulcers. These were considered specific features of CMV infection. Endoscopic ulcers that did not fit these categories were defined as irregular ulcers. Each endoscopic image was evaluated by two authors (Kazuki Kakimoto and Takuya Inoue).

### Statistical analysis

Demographic and clinical parameters were compiled and summary statistics were calculated. Qualitative data were compared using Mann-Whitney’s *U* test and Fisher’s exact test or Chi-squared test, as appropriate. A univariate analysis was performed using logistic regression to determine the independent effects of variables that were associated with CMV infection. Subsequently, a multiple logistic regression analysis was performed for variables with a *p* value <0.15 in the univariate analysis, based on the backward Wald selection method. The results are reported as an odds ratio (OR) with 95% confidence interval (95% CI), or as a mean ± SD; a *p* value <0.05 was considered statistically significant. All calculations were performed using the Statview system (SAS Institute, Cary, NC).

## Results

### Patient characteristics

Eighty-six patients with exacerbated UC who were admitted to our hospital were consecutively enrolled. Table 1 shows the clinical characteristics and treatments that the patients received at the time they were included in the study. Of these 86 patients, 26 (30.2%) had positive CMV DNA qPCR results; only 4 of these patients were also positive for antigenemia (Table 2). No significant differences were found for clinical characteristics such as sex, disease duration and extent, steroid resistance and dependence, and C-reactive protein level between CMV DNA-positive and CMV DNA-negative patients. However, CMV DNA-positive patients were significantly older than CMV DNA-negative patients (*p* = 0.002) (Table 1). The mean endoscopic score of patients who were positive for CMV infection was significantly higher than that of patients who were negative for CMV infection (7.3 ± 1.1 and 6.4 ± 1.5, respectively; *p* = 0.016).

The 14 patients who were treated with monotherapy included 5 who received tacrolimus, 4 who received biologics, and 5 who received thiopurine. Combination therapy was used significantly more frequently by CMV DNA–positive patients than by CMV DNA-negative patients (*p* = 0.038); only 5-ASA treatment was used significantly more often by CMV DNA-negative patients (*p* = 0.037).

### Risk factors for CMV infection

Table 3 shows the risk factor analysis for CMV infection. Age was significantly associated with a higher risk of positive CMV DNA results than other factors (OR: 1.083; 95% CI: 1.025–1.143; *p* = 0.0041). The univariate analysis showed that there were positive associations between a high score (>7) of Blackstone’s endoscopic score and high risk for CMV infection (*p* = 0.0467). However, the multiple conditional logistic regression analysis to account for potential confounding variables found that there was no association between a high endoscopic score and the risk for CMV (*p* = 0.1655). Specific endoscopic features of CMV were not significantly associated with negative results for CMV infection (OR: 0.077; 95% CI: 0.012–0.492; *p* = 0.0068); punched-out ulcers and wide mucosal defects were not correlated with positive CMV DNA (*p*>0.05). Combination therapy was associated with a higher risk of positive CMV DNA results (OR: 7.439; 95% CI: 1.001–55.304; *p* = 0.0499). Treatment with 5-ASA alone, PSL, and monotherapy was not associated with a higher risk of CMV infection.

### Outcomes of UC patients who were positive for CMV DNA in the colonic mucosa

Of 26 patients who had positive qPCR results for CMV DNA in the colonic mucosa, 19 were treated with oral tacrolimus, 2 were treated with infliximab, and 2 was treated with prednisolone as an additional therapy after hospital admission. Twenty-two of these 26 patients (84.6%) responded to the additional therapy within 1 month and only 1 patient underwent colectomy. All patients who were positive for antigenemia were treated concomitantly with ganciclovir. Fourteen of 15 (93.3%) patients showed a clinical response after treatment with additional oral tacrolimus without ganciclovir. Three of 4 patients responded to the oral tacrolimus with concomitant ganciclovir.

**Fig. 2:**
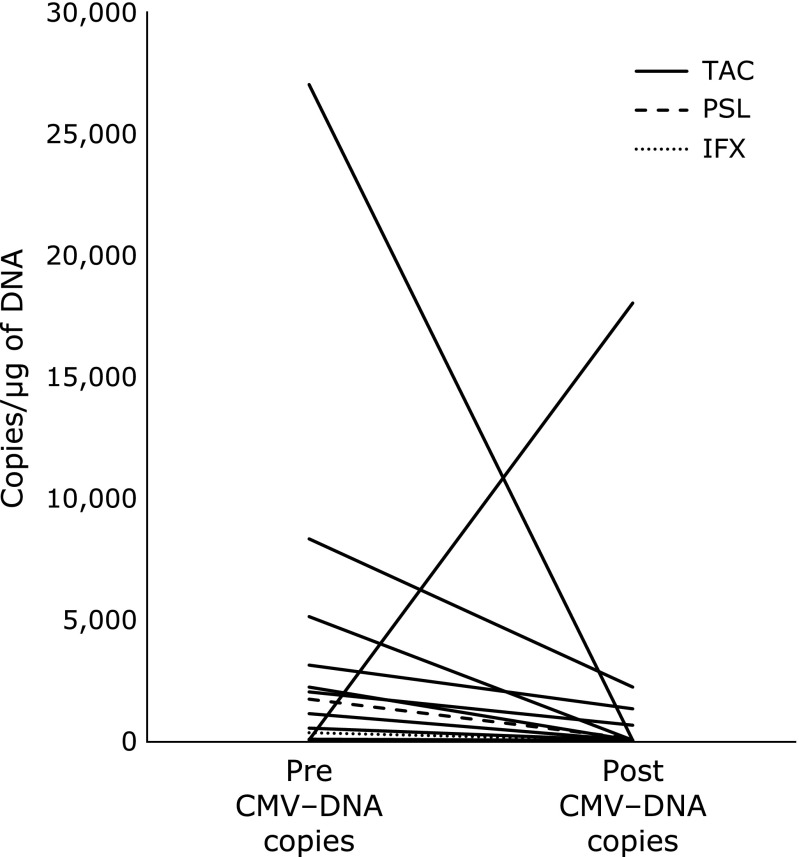
The number of cytomegalovirus (CMV)-DNA copies before and after the treatment of active UC patients with CMV infection (mean duration between colonoscopies: 21.3 ± 11.2 days).

Fifteen patients underwent repeated colonoscopies after the additional drug therapy. Then, qPCR was performed to determine the CMV DNA of the colonic mucosa again (mean duration between colonoscopies: 21.3 ± 11.2 days) (Fig. 2). Of these 15 patients, 13 were treated with tacrolimus. Twelve of these 13 patients (92.3%) showed a decrease in the number of CMV DNA copies compared with the pre-treatment levels.

## Discussion

CMV infection was reported to cause refractory UC.^(1,2)^ Because the specific endoscopic features of refractory UC that is associated with CMV infection have not been clearly delineated, diagnosing CMV infection at an early stage is difficult.^(6)^ During this study, we used qPCR to identify patients with exacerbated UC and concomitant CMV infection, and assessed the risk factors and outcomes of patients with CMV infection. Our results show that treatment with combined immunosuppressants, including PSL, was a risk factor for CMV infection. Furthermore, the majority of CMV DNA-positive patients who were treated with additional oral tacrolimus without ganciclovir had clinical responses and decreased CMV DNA copy numbers in their colonic mucosa. To our knowledge, this is the first report describing the effects of oral tacrolimus on patients with exacerbated UC who have positive CMV DNA.

Several studies have been conducted to identify the characteristic endoscopic features of UC patients with CMV infection. Few reports showed that certain endoscopic findings, such as punched-out ulcers, are characteristic of UC that is associated with CMV infection.^(19,21)^ In contrast, many studies have failed to elucidate the specific endoscopic features.^(22–24)^ In the current analysis, although the absence of longitudinal ulcers and irregular ulcers was associated with negative results for CMV infection, the characteristic endoscopic features of patients with exacerbated UC that was associated with positive qPCR results for CMV DNA were not found. Because the endoscopic features of refractory UC are similar to those of UC that is concomitant with CMV infection,^(20)^ we consider it difficult to diagnose UC that is associated with CMV infection based solely on endoscopic features.

CMV DNA was detected in the colonic mucosa of 26 of 86 patients (30.2%). Among these 26 patients, only 4 had positive antigenemia assay results, even though the CMV DNA copy numbers were relatively high in some patients. Interestingly, the majority of CMV DNA-positive patients had clinical responses and decreased CMV DNA copy numbers after additional treatment without concomitant ganciclovir. Furthermore, the majority of these patients were treated with oral tacrolimus, and CMV reactivation might be induced by immunosuppressive drugs such as corticosteroids and immunomodulators. Therefore, patients with UC are at a high risk for developing CMV infection.^(22)^ For example, cyclosporine A (CsA) has been reported to enhance the replication of CMV.^(5)^ Tacrolimus is a calcineurin inhibitor with activity that is similar to that of CsA, including suppression of mixed lymphocyte reactivity and interference with the production of interleukin 2.^(22,25,26)^ However, in a randomized trial comparing tacrolimus and CsA to prevent liver allograft rejection, the incidence of CMV infection was significantly lower for patients receiving tacrolimus than for those receiving CsA (15.7% and 25.0%, respectively).^(14)^ It has also been reported that tacrolimus has a suppressive *in vitro* effect on CMV replication and a suppressive *in vivo* effect on intracellular virus growth at the concentration that is used for immunosuppression.^(16)^ Alessiani *et al.*^(15)^ also reported that tacrolimus treatment for liver transplant recipients resulted in a significantly lower incidence of symptomatic CMV infection than that observed after CsA treatment. Because the early diagnosis of CMV infection is still difficult in patients with refractory UC, clinicians are often faced with the difficult decision to initiate antiviral therapy or additional immunosuppression. Based on the results of this study, we believe that starting with oral tacrolimus may be a useful strategy for patients with severe or refractory UC.^(27,28)^

This study has several limitations. First, of the 26 patients with positive qPCR results for CMV DNA in the colonic mucosa, only 15 underwent repeated qPCR to detect CMV DNA after additional therapy was administered. Regarding the other 11 patients who were not followed-up using qPCR, 8 showed a clinical response and 1 underwent colectomy due to fulminant colitis. Second, combination therapy included several combinations of immunomodulators. Third, of the 26 patients with positive qPCR results for CMV DNA, 4 were treated with additional therapy that did not include tacrolimus (infliximab and PSL). Of these 4 patients, 3 showed a clinical response without ganciclovir. This means that several therapies other than tacrolimus may be useful for treating CMV infection if these therapies are effective against UC itself. Infliximab was reported to be effective against CMV infection.^(8)^ Fourth, it is unknown whether patients who were negative for antigenemia and cytomegalovirus DNA-positive colonic mucosa could be cured only by ganciclovir. Finally, this study was retrospective; therefore, bias due to patient collection may inevitably exist.

In conclusion, we used qPCR analysis to identify patients with CMV infection and active UC, and assessed the risk factors that are associated with positive qPCR results for CMV DNA in the colonic mucosa. Because additional treatment with oral tacrolimus was effective, patients who are negative for antigenemia and have CMV DNA-positive colonic mucosa may recover without needing antiviral therapy.

## Figures and Tables

**Table 1:** Clinical characteristics of all patients and the CMV-DNA positive and negative subgroups

	All patients (*n* = 86)	CMV (+) (*n* = 26, 30.2%)	CMV (–) (*n* = 60, 69.8%)	*p*
Mean age (years) ± SD	47.4 ± 16.5	55.0 ± 13.2	41.8 ± 16.2	0.002
Male	53 (61.6%)	15 (17.4%)	38 (44.2%)	0.801
Female	33 (38.4%)	11 (12.8%)	22 (25.6%)	
Duration of disease(month)	76.3 ± 78.7	71.4 ± 60.8	80.0 ± 89.4	0.687
Extention				
Total colitis	78 (90.7%)	24 (27.9%)	54 (62.8%)	0.948
Left side	7 (8.1%)	2 (2.3%)	5 (5.8%)	0.742
Steroid				
Resistance	17 (19.8%)	8 (9.3%)	9 (10.5%)	0.164
Dependent	17 (19.8%)	4 (4.7%)	13 (15.1%)	0.706
CRP	5.0 ± 6.3	5.0 ± 5.1	5.1 ± 7.1	0.947
Endoscopic score	6.8 ± 1.4	7.3 ± 1.1	6.4 ± 1.5	0.016
Treatment				
5-ASA	36 (41.9%)	6 (7.0%)	30 (34.9%)	0.037
+PSL	22 (25.6%)	9 (10.5%)	13 (15.1%)	0.32
+Mono	14 (16.3%)	3 (3.5%)	11 (12.8%)	0.641
+Combo	14 (16.3%)	8 (9.3%)	6 (7.0%)	0.038

**Table 2:** Clinical characteristics of patients with CMV infection

No.	Age/Sex	Extension	Endoscopic score	CMV–DNA Copies	Antigenemia	PSL (mg)	TAC (mg)	Bio	IM (mg)	Group
1	79 F	Left sided	8	46	–	–	–	–	–	5ASA
2	71 M	Total	8	240	–	–	–	–	–	5ASA
3	26 F	Total	8	300	–	10	–	–	–	PSL
4	54 M	Total	8	510	–	10	–	–	–	PSL
5	33 F	Total	8	1,100	–	20	–	IFX	AZA 50	Combo
6	54 M	Total	8	1,600	–	–	–	–	–	5ASA
7	64 M	Total	6	2,000	–	–	–	–	AZA 50	Mono
8	62 F	Left sided	8	3,100	–	30	–	–	–	PSL
9	68 M	Total	8	5,100	–	5	–	–	–	PSL
10	47 M	Total	8	27,000	–	40	–	–	–	PSL
11	50 M	Total	5	83,000	–	–	–	IFX	AZA 50	Combo
12	39 M	Total	8	16	–	20	–	–	AZA 50	Combo
13	55 F	Total	8	68	–	–	–	–	–	5ASA
14	38 M	Total	8	80	–	20	–	–	–	PSL
15	54 M	Total	8	21	–	50	–	–	–	PSL
16	55 M	Total	5	11,000	–	–	–	–	AZA 50	Mono
17	41 F	Total	7	14	–	–	–	–	–	5ASA
18	81 M	Total	7	1,700	–	–	–	–	–	5ASA
19	40 M	Total	7	320	–	–	2	–	AZA 50	Combo
20	59 F	Total	5	570	–	20	4	–	–	Combo
21	60 F	Total	7	10,000	–	–	2	IFX	–	Combo
22	60 F	Total	6	17,000	–	–	4	ADA	–	Combo
23	51 M	Total	6	260	+	–	2	–	–	Mono
24	68 M	Total	8	2,200	+	30	–	–	–	PSL
25	56 M	Total	8	64,000	+	30	–	–	–	PSL
26	65 M	Total	8	99,000	+	15	–	IFX	AZA 50	Combo

**Table 3:** Univariate and multivariate logistic regression models to evaluate risk factors for CMV infection with UC

		Univariate model				Multivariate model		
		OR	95% CI	*p*		OR	95% CI	*p*
Age	Years	1.06	1.018–1.103	0.0045		1.083	1.025–1.143	0.0041
Sex	Male	0.963	0.329–2.815	0.9444				
Duration of disease	Month	0.988	0.912–1.071	0.7697				
Extention	Total colitis	0.375	0.032–4.382	0.4342				
Steroid	Resistance	2.133	0.597–7.625	0.2436				
	Dependent	0.571	0.146–2.234	0.4211				
CRP	mg/L	1.005	0.926–1.091	0.9054				
Endoscopic score	>7	3.111	0.968–9.999	0.0467		2.8	0.653–11.996	0.1655
Endoscopic features	Longitudinal ulcers	–	–	–				
	Punched-out ulcers	5.091	0.530–48.863	0.1584				
	Wide mucosal defects	2.671	0.687–10.386	0.1562				
	No specific features	0.153	0.044–0.527	0.0029		0.077	0.012–0.492	0.0068
Treatment	5-ASA	0.321	0.100–1.033	0.0567		0.422	0.082–2.169	0.3015
	+PSL	2.029	0.606–6.795	0.251				
	+Mono	0.626	0.134–2.925	0.5516				
	+Combo	3.852	0.897–16.533	0.0696		7.439	1.001–55.304	0.0499

## Abbreviations

5-ASA: 5-aminosalicylic acid
95% CI: 95% confidence interval
CMV: cytomegalovirus
CRP: C-reactive protein
CsA: cyclosporine A
OR: odds ratio
PSL: prednisolone
qPCR: quantitative real-time polymerase chain reaction
UC: ulcerative colitis
